# Can low autopsy rates be increased? Yes, we can! Should postmortem examinations in oncology be performed? Yes, we should! A postmortem analysis of oncological cases

**DOI:** 10.1007/s00428-020-02884-8

**Published:** 2020-07-10

**Authors:** Johanna Waidhauser, Benedikt Martin, Martin Trepel, Bruno Märkl

**Affiliations:** 1 Institute of Pathology and Molecular Diagnostics, University Medical Center Augsburg, Stenglinstraße 2, 86156 Augsburg, Germany; 2 Department of Hematology and Medical Oncology, University Medical Center Augsburg, Stenglinstraße 2, 86156 Augsburg, Germany

**Keywords:** Autopsy, Postmortem examination, Rate, Tumor, Oncology, Discrepancy

## Abstract

Ever declining autopsy rates have been a concern of pathologists as well as clinicians for decades. Notably, in the field of oncology, data on autopsies and discrepancies between clinical and autoptic diagnoses are particularly scarce. In this retrospective study, we show the effect of a simple catalog of measures consisting of a different approach to obtain consent for autopsy, structured conferencing, and systematic teaching of residents, as well as a close collaboration between clinicians and pathologists on the numbers of autopsies, especially of oncological patients. Additionally, postmortem examination protocols from the years 2015 until 2019 were analyzed, regarding rates of discrepancies between clinical and autoptic causes of death in this category of patients. Autopsy numbers could be significantly increased from a minimum in 2014 (60 autopsies) to a maximum in 2018 (142 autopsies) (*p* < 0.0001). In the 67 autopsies of oncological cases, a high rate of 51% of major discrepancy between clinical and autoptic causes of death could be detected. In contrast to the general reported decline of autopsy rates, we present rising autopsy numbers over the past 5 years with an increasing number of oncological cases who underwent a postmortem examination. The high percentage of major discrepancies between clinical and autopsy diagnosis is in contrast to an expected decrease of major discrepancies in times of precise diagnostic methods and underlines the importance of autopsies to ensure high quality in diagnostics and therapy not only in the field of oncology.

## Introduction

Autopsy rates have been decreasing for several decades. Although general registries for autopsy rates are lacking, declining numbers of postmortem examinations have been reported in numerous publications. In Germany, a decline in overall numbers of autopsies of 30% between 2005 and 2014 was detected [1]. This number is concordant with previous reports from other countries such as France, Sweden, and the USA where the decline has been reported to be even more severe [2, 3]. A survey among pathologists in Germany revealed an autopsy rate of patients who died in a hospital of 3.63% in 2013 and 3.39% in 2014 [4]. Numerous reasons are quoted for the decline. Besides costs and cultural reasons, differing working patterns of pathologists with focus on surgical pathology and considerations about the necessity of postmortem examinations in times of improved radiological and endoscopic diagnostics play an important role [5–7]. The decrease in autopsy numbers is not only a recent phenomenon. Already in 1983, declining autopsy rates were attributed to diagnostic progress, resulting in a presumably decreasing rate of discrepancies between clinical judgment and autopsy findings. Of note, however, this decline in discrepancies was not detectable in a large analysis by Goldman et al. [8]. In the subsequent almost 30 years, medical progress has accelerated considerably. In recent publications however, the rate of major discrepancies remains considerably high with 19.5–28% [3, 9–11]. In oncological patients, detailed studies of postmortem examinations are lacking. The few existing investigations are mainly on cancer patients who died at intensive care units and show rates of major discrepancies between 26 and 59% [12, 13]. In this retrospective analysis, we want to demonstrate how low autopsy rates can be increased by optimized structured interaction of pathologists and clinicians. Furthermore, we want to evaluate the relevance of autopsies of oncological patients.

## Methods

### Means to increase autopsy rates

In 2015, at the beginning of the analyzed period, a catalog of measures was established in the II. Department of Internal Medicine in order to increase the number of autopsies. Deaths had to be reported in the morning meeting the next day. Residents were instructed with regard to structured conversations with relatives. This instruction took place a first time in 2015 and was repeated regularly afterwards in the morning conferences in association with the discussion of decedents and the consent to autopsy. Furthermore, a demonstration of autoptic findings to clinicians took place after every autopsy and a weekly demonstration of relevant cases was given by the pathologists in front of the whole team of the II. Department of Internal Medicine.

### Analysis of oncological cases

Annual autopsy numbers of adult patients of the past decade were retrieved from the local database at University Medical Center Augsburg. Patients who died in the II. Department of Internal Medicine, which comprises hematology and medical oncology as well as nephrology and an intensive care unit, from January 2015 to October 2019 were screened for known and active oncological diagnoses at the time of death by checking the medical record. Incomplete autopsies as well as incidentally found and not previously suspected tumor diagnoses were excluded. Of the remaining cases, demographic data, oncological diagnoses, clinically assumed cause of death, the specific question of clinicians to pathologists for autopsy, and the autoptically revealed cause of death were retrieved from the postmortem examination protocol. The information on the clinical cause of death were reported in the autopsy protocol according to the death certificate, the medical information in the autopsy request and the physician’s letter. Additionally, the treating clinician gave an overview of the case in the course of the presentation of the autoptic findings. In unclear cases, the electronic file of the patient was consulted by the authors. The cause of death revealed by autopsy was separated into immediate cause of death, underlying diagnoses, and basic diagnoses in the autopsy protocol. For our analysis however, only the immediate cause of death was considered. If necessary, the reported results were interpreted by the authors to state one immediate cause of death. Subsequently, discrepancies between clinical and autoptic diagnosis were categorized according to the discrepancy criteria displayed in Table 1***.*** In the special situation of oncological patients, every major diagnosis was individually assessed for its treatability and therefore classified as class I or class II discrepancy. For each case, the highest level of discrepancy was recorded as well as further levels of discrepancy if applicable. Unclear cases were discussed with the head of the Department of Pathology and Molecular Diagnostics (BM). Only diagnoses that were detected or confirmed during autopsy were taken into account. Therefore, false-positive clinical diagnoses were not included. Main diagnoses were categorized into seven thematic fields: infection/ sepsis, embolism, cardiac failure (comprising deaths of ischemic cardiac failure and non-ischemic cardiac failure, for example, deaths of hypertensive heart disease, arrhythmic events, or pericardial tamponade), tumor (for example, spreading to vital organs or perforation of hollow organs due to the tumor), bleeding, others (for example, liver failure as a result of the tumor therapy, ileus, thrombotic microangiopathy), and unknown cases.

**Table 1:** Classification of discrepancies according to Goldman [8] and Battle [14]

	Level of discrepancy	Description	Example
**Major discrepancy**	Class I	Missed treatable primary diagnosis with direct correlation to cause of death. Detection and treatment would in all probability have led to prolonged survival.	Missed myocardial infarction without cardiac arrest or undetected gastrointestinal bleeding with therapeutical option.
	Class II	Missed primary diagnosis with unclear impact on survival. Detection would probably not have resulted in a changed treatment (for example because of lacking treatment options).	Perforation of the stomach without therapeutical option or unknown spreading of the tumor to vital organs under tumor therapy.
**Minor discrepancy**	Class III	Missed secondary diagnosis with no direct correlation to cause of death but symptoms that would have indicated treatment. Detection and treatment would probably have an impact on prognosis.	Unknown arteriosclerosis or old myocardial infarction without prior treatment.
	Class IV	Missed secondary occult diagnosis with no indication for treatment but of possible genetic or epidemiological importance.	Asymptomatic cholecystolithiasis or struma.
	Class V	No discrepancy.	
	Class VI	Unclear cases.	

The study was approved by the local medical ethical committee (institutional review board, reference number BKF 2019/35).

Statistical analyses were performed with SigmaPlot 13.0 (Systat, Erkrath, Germany) and Microsoft Excel (Microsoft Office 16). Chi-square test was used to compare frequencies of major discrepancies between male and female as well as old and young patients, patients who did or did not receive immunotherapy, and between early years of analysis and later years. Two tailed *t*-test was performed for comparing continuous normally distributed data. A *P* value of <0.05 was considered significant.

## Results

### Increase of autopsy numbers

**Fig. 1:**
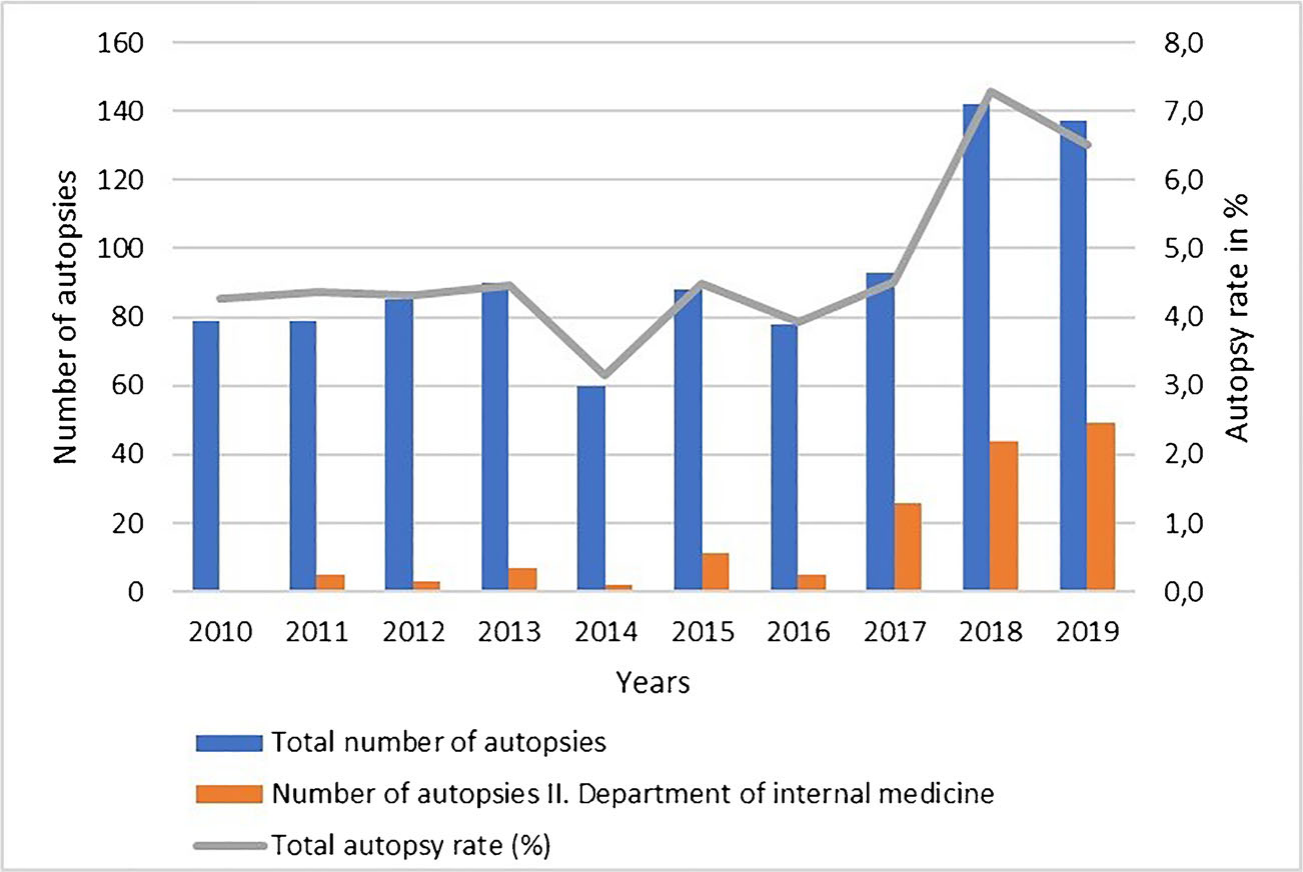
Numbers and rates of autopsies conducted between 2010 and 2019 at the University Medical Center Augsburg

Annual autopsy numbers varied between 60 and 93 in the years 2010 until 2017 and showed a significant increase (*p* < 0.0001) in 2018 and 2019, with 142 and 137 postmortem examinations performed at the University Medical Center Augsburg. Of these, the fraction of autopsies of patients who died in the II. Department of Internal Medicine rose from 0% in 2010 to 35.8% in 2019. The total number of patients who deceased in the University Medical Center of Augsburg during this time period varied between 1.850 and 2.083 per year with a median of 1963. The overall autopsy rate was calculated with a minimum of 3.1% in 2014 and a maximum of 7.3% in 2018. Numbers and rates of autopsies of the years 2010–2019 are shown in Fig. 1***.***

### Results of oncological autopsies

**Fig. 2:**
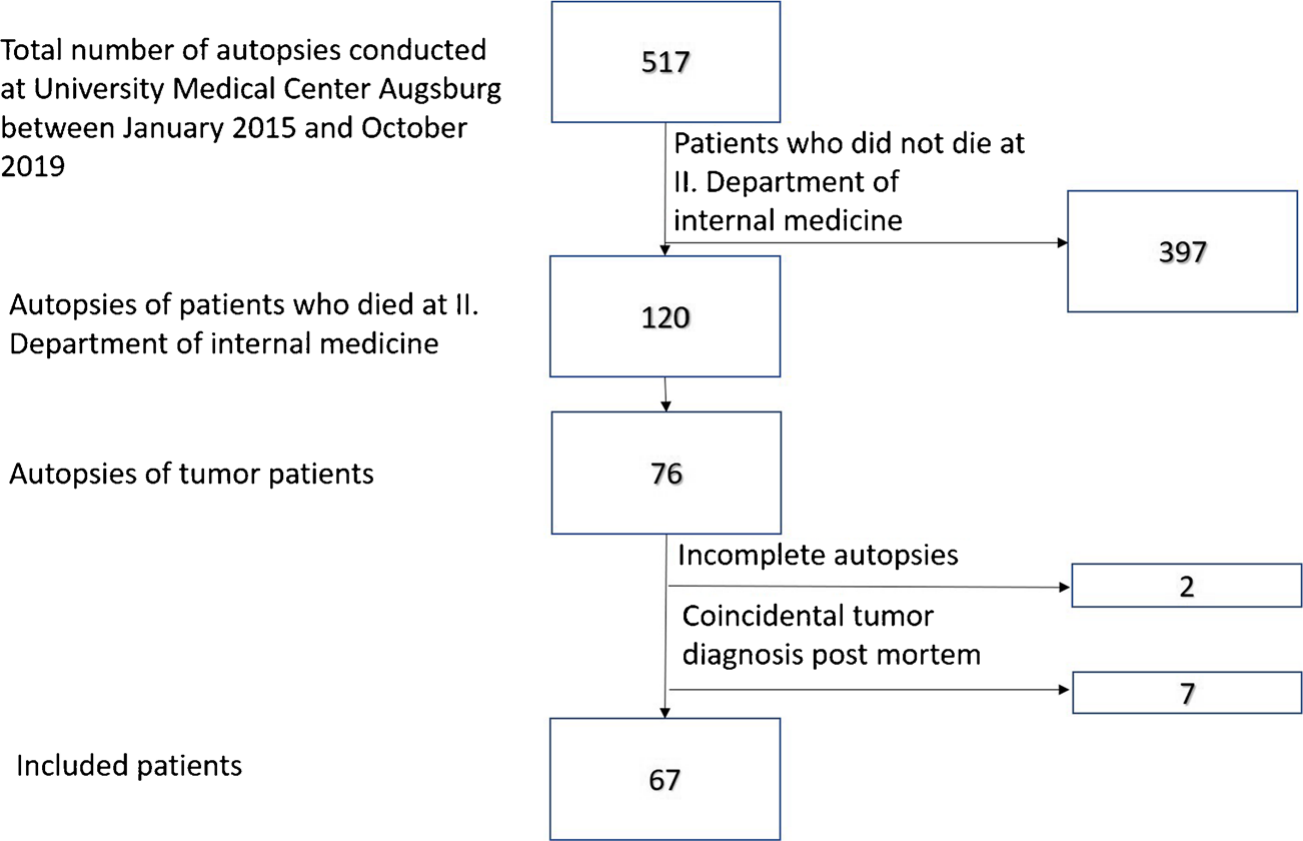
Selection process of oncological cases

Between January 2015 and October 2019, a total of 517 autopsies were performed in the Department of Pathology. Of these, 120 were cases that were previously treated at the II. Department of Internal Medicine and in 76 of these, a tumor diagnosis was reported in the medical record. After removing cases in which the tumor diagnosis was not known before and made incidentally during the postmortem examination and incomplete autopsies of for example only bone marrow or brain, 67 cases of patients with a known and active tumor disease were eligible for the analysis (Fig. 2).

Demographic and disease characteristics are shown in Table 2***.*** The median age was 67 (24–93) years. Interestingly, 48 (72%) patients were male and 19 (28%) female (*p* = 0.028). The most frequent tumor entities were of the blood and lymphatic system (37%) and of the lung (22%). In total, 84% of the patients had died at an advanced tumor stage and 10% of the patients had been previously treated with an immunotherapy.

**Table 2:** Demographic and disease characteristics

Variables	Results
Age; *median (range)*	67 (24–93)
Sex	
Male; *n (%)*	48 (72)
Female; *n (%)*	19 (28)
Tumor type	
Gastrointestinal tract; *n (%)*	4 (6)
Liver/gall bladder/pancreas; *n (%)*	6 (9)
Urogenital tract; *n (%)*	5 (7)
Female genital tract; *n (%)*	1 (1)
Breast; *n (%)*	5 (7)
Blood/lymphatic; *n (%)*	25 (37)
Head and neck; *n (%)*	2 (3)
Lung; *n (%)*	15 (22)
Skin/melanoma; *n (%)*	4 (6)
Brain; *n (%)*	1 (1)
Other; *n (%)*	1 (1)
Stage	
Advanced; *n (%)*	56 (84)
Limited; *n (%)*	11 (16)
Immunotherapy	
Yes; *n (%)*	7 (10)
No; *n (%)*	60 (90)

The clinically assumed cause of death was infection with sepsis in the majority of cases which was consistent with autoptic findings. Embolism, tumor-associated causes of death, and cardiac failure were more frequently found in postmortem examinations than clinically suspected *(*Table 3)**.**

**Table 3:** Comparison of clinical and autoptic immediate cause of death

Clinical cause of death	Autoptic cause of death						
	Infection/ Sepsis	Embolism	Cardiac failure	Tumor	Bleeding	Other	Unknown
Infection/sepsis (*n* = **23**)	**18**	1	0	1	0	1	2
Embolism (*n* = **4**)	0	**1**	1	0	0	1	1
Cardiac failure (*n* = **2**)	0	0	**0**	2	0	0	0
Tumor (*n* = **7**)	0	1	1	**4**	0	0	1
Bleeding (*n* = **2**)	0	0	0	0	**2**	0	0
Other (*n* = **15**)	2	1	0	3	0	**9**	0
Unknown (*n* = **16**)	4	2	5	2	1	1	**1**

The specific question addressed to the pathologist was “cause of death” in 48% of cases, followed by questions concerning the tumor disease like specific histology or tumor extension/stage.

**Fig. 3:**
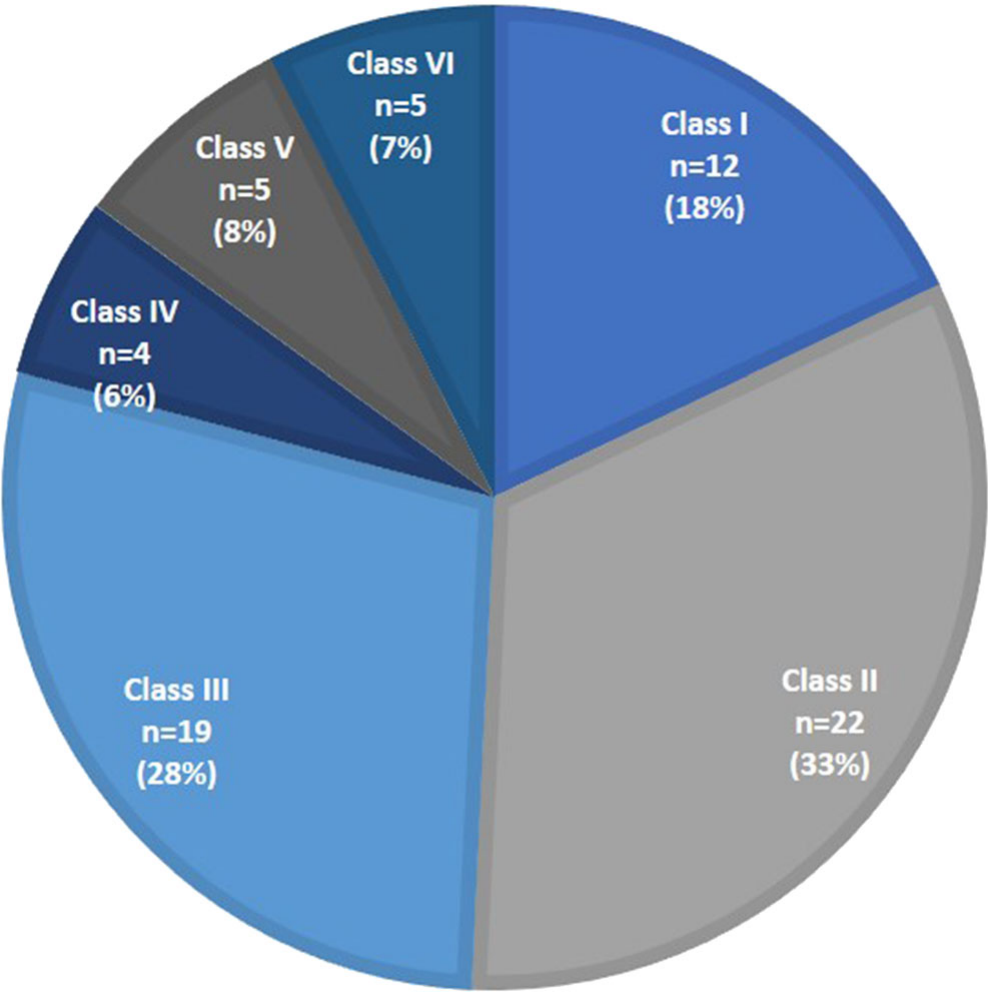
Highest documented level of discrepancy between clinical and autoptic diagnosis. Class I and II: major discrepancies. Class III and IV: minor discrepancies. Class V: no discrepancy. Class VI: unclear cases

**Fig. 4:**
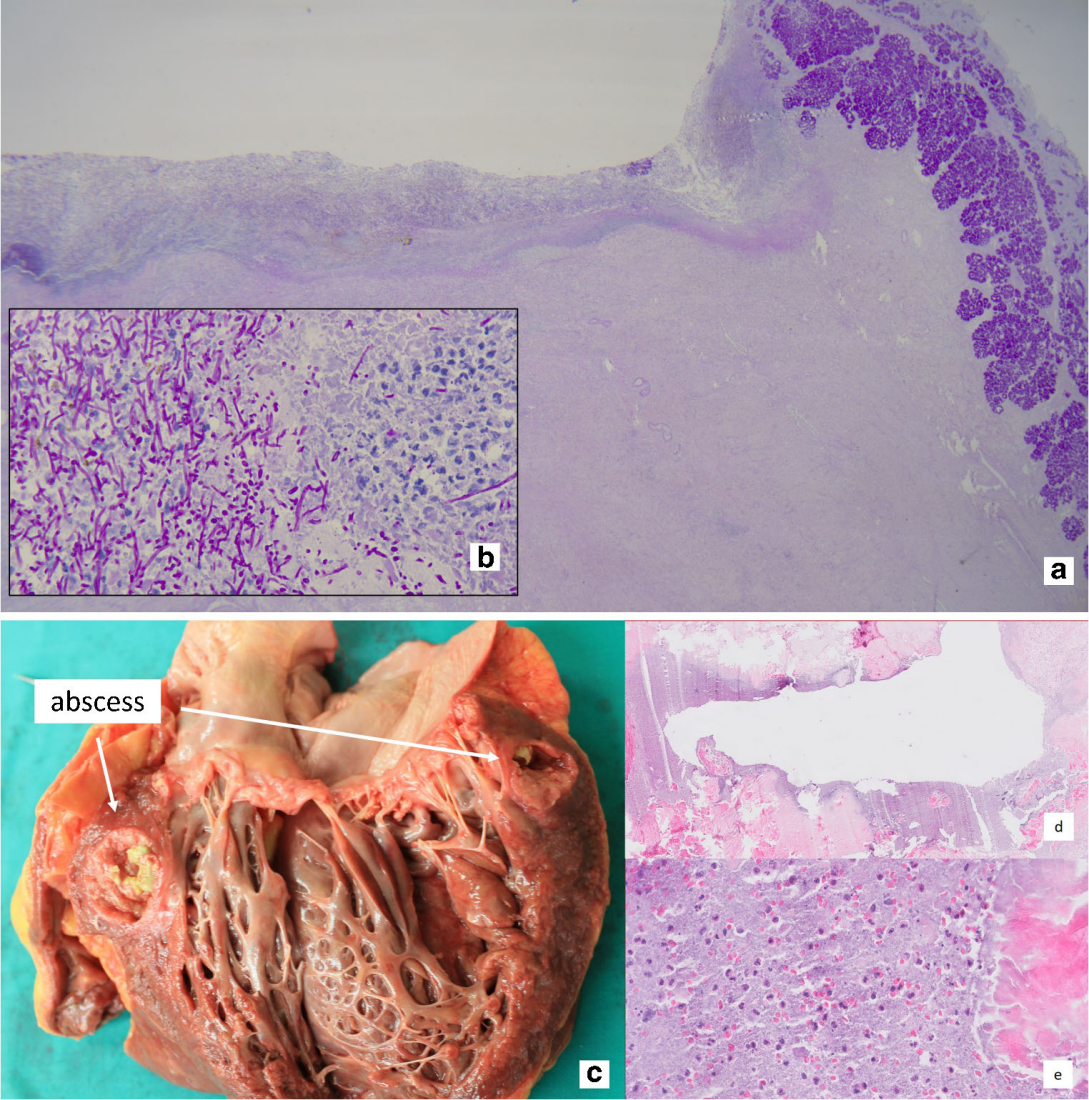
Examples for major discrepancies. **a**, **b** Class I discrepancy: patient with a seminoma and an unknown upper gastrointestinal bleeding due to a duodenal ulcer on the basis of a mycosis (**a** histological slice (PAS) overview (12,5); **b** histological slice (PAS) detailed enlargement (630)). **c**, **d**, and **e** Class II discrepancy: previously unknown myocardial abscess of the mitral perivalvular region with proof of a vancomycin-resistant enterococcus (VRE) in a patient with MDS (**c** overview of the abscess; **d** histological slice (H&E), overview; **e** histological slice (H&E) detailed enlargement with granulocyte infiltration, debris, and calcifications)

For each case, the highest level of discrepancy was documented. In 51% of autopsies, major discrepancies between clinical and postmortem assessment were detected (18% class I and 33% class II). Only in 8% of cases, neither major nor minor discrepancy was found (Fig. 3). The most frequent undetected diagnoses that were classified as class I discrepancy were infection/sepsis (*n* = 6) and pulmonary embolism (*n* = 4). Table 4 gives an overview of the clinical and autoptic cause of death of the 12 patients with a class I discrepancy. Undetected diagnoses that were classified as class II discrepancy included infection/sepsis (*n* = 6), cardiac failure (*n* = 6), tumor-related diagnoses (*n* = 6), embolism (*n* = 2), and others (*n* = 2). Figure 4 shows exemplary cases for discrepancies class I and class II.

**Table 4:** Clinical and autoptic causes of death of patients with class I discrepancy. *NSCLC* non-small cell lung cancer, *CMML* chronic myelomonocytic leukemia, *AML* acute myeloid leukemia

**Nr.**	**Tumor entity**	**Clinical cause of death**	**Autopsy cause of death**
3	NSCLC undifferentiated	Unknown	Pneumonia, sepsis
5	NSCLC poorly differentiated,	Unknown, reduced vigilance	Myocardial infarction (previously unknown coronary heart disease) with cardiovascular failure
13	CMML	Pneumonia	Septic/toxic cardiovascular failure, due to superinfection of skin lesions; previous myocardial damage due to 2–4 weeks old myocardial infarction
14	Multiple Myeloma	Unknown	Septic/toxic cardiovascular failure with multiple septic spots of unknown origin in the lung
15	Mamma carcinoma	Unclear sedation and seizure	Septic shock with multiorgan failure due to pneumonia
17	Merkel cell carcinoma	Aspiration	Pulmonary embolism, central occlusion of the left pulmonary artery
20	Seminoma	Unknown	Upper gastrointestinal bleeding due to duodenal ulcer due to Mycosis
29	Pancreas carcinoma	Pneumonia, hematuria	Respiratory failure due to multiple pulmonary embolisms with right heart overload
33	AML	Respiratory failure or cerebral bleeding	Sigma diverticulosis with abscessed inflammation and urinary bladder fistula
35	Cholangiocellular carcinoma	Multiorganfailure due to tumor progression	Bilateral pulmonary embolism with right heart overload
45	Prostate carcinoma	Sepsis, acute kidney failure	Pulmonary embolism with right heart overload
57	Small cell carcinoma of the liver	Pneumonia	Aortal valve endocarditis with sepsis

Regarding secondary findings, the most frequent class III discrepancies were arteriosclerosis (*n* = 9), pulmonary emphysema (*n* = 8) and small or old myocardial infarctions (*n* = 4). The most frequent class IV discrepancies were struma (*n* = 12), benign changes of the female genital tract like uterus myomatosus or fibroma/ cystadenoma of the ovary (*n* = 7), and diverticulosis (*n* = 7).

In comparing frequencies of major discrepancies between male and female patients, no significant difference could be detected using chi-square test (*p* = 0.15). Likewise, no significant difference of major discrepancies could be found in younger and older patients (*p* = 0.38), in earlier years with lower autopsy rates (2015–2017) compared to later years with increased autopsy rates (2018/2019) (*p* = 0.54), in patients with advanced tumor disease compared to patients with limited disease (non-metastatic, no local invasion of surrounding tissue) (*p* = 0.35) or in patients who did or did not receive immunotherapy (*p* = 0.72).

## Discussion

### Effect of the measures to increase autopsy rates

In contrast to the generally reported decline of autopsy numbers, increasing rates of autopsies could be seen at the University Medical Center Augsburg since 2017. These were mostly attributed to a rising number of patients premortally treated at the II. Department of Internal Medicine including oncological cases. The increase of autopsy numbers could be achieved with a simple catalog of measures as described in the “Methods” part. As main reasons for the successful change in autopsy policies, an increased awareness of the topic, as well as an intensive cooperation between clinicians and pathologists not only in everyday work but also in research and teaching, can be named. The motivation of clinical residents of the II. Department of Internal Medicine to address the topic of postmortem examinations with patients and relatives could be augmented. Furthermore, the manner in which the topic was addressed changed from an open question to a precise proposal with explanations regarding the benefits of an autopsy. The fact that communication with patients and relatives plays an important role to establish a positive view on autopsies and to obtain consent for the procedure was shown by Rosenberg et al. [15]. In this study, motivational interviewing of patients and relatives led to a significant increase of postmortem examinations. The quality of interaction between clinicians and pathologist as an approach to higher autopsy numbers was described previously by Champ et al. who identified regular meetings between pathologists and clinicians and a positive attitude towards the other discipline respectively, to play an important role [16]. In our institution, the combination of both named approaches resulted in a significant increase of postmortem examinations. An additional concept to increase autopsy numbers has been proposed by Van den Tweel and Wittekind, suggesting the creation of “autopsy pathology as a subspecialty” to overcome the problem of work overload for pathologists and the problem of late and in parts also insufficient reporting of postmortem examinations [6].

### Relevance of autopsies in oncological patients

Regarding the demographic data of the deceased patients, a significantly higher number of male patients underwent a postmortem examination. These findings are consistent with previous publications [3]. According to Cameron et al., the reason for higher autopsy rates in male patients is the higher likelihood of female relatives to give the permission for a postmortem examination [17].

The most frequent question to the pathologist in our study was “cause of death” with 48%, followed by questions with relation to the tumor disease. Previous studies analyzing questions addressed to postmortem examinations are scarce. In a study by Zarbo et al., “cause of death” was the question in 21% and tumor-specific questions were asked only in 2% of cases [18]. However, it has to be taken into account that the population of this study comprised various primary diagnoses while in our study population every patient had an oncological diagnosis.

In this retrospective analysis, a considerably high number of major discrepancies between clinical and postmortem diagnoses could be found. In previous studies and reviews, rates for major discrepancies varied between 4.1 and 49.8% with the highest rates in postoperative patients [19]. An assumed decline of discrepancy rates over the years due to improved premortal diagnostic technical potential could not be verified by Goldman et al. in 1983 [8] and in further analyses comparing different time periods [20, 21]. In contrast, a systematic review by Shojania et al. described a decline of major discrepancies of 19.4% per decade in 53 autopsy series over 40 years. The authors explained the difference between lacking declines in single institution analyses and clear reduction of discrepancies in their review with the insufficient power of the single-center studies and stricter selection of cases in times of declining autopsy rates [19]. In the relatively short time period considered in our study, no significant difference of major discrepancy rates between the earlier years (2015–2017) and later years (2018 and 2019) could be seen.

In addition to the time period in which an autopsy was performed, decreasing autopsy rates were linked to higher discrepancy rates in the mentioned review [19]. The consideration that increased autopsy rates lead to lower selection bias towards unclear cases could serve as a possible explanation for this observation. This phenomenon, however, could not be observed in our study population where the numbers of class I and II discrepancies showed no significant change in the years with higher autopsy rates.

Gender and age of decedents did not play a role for the frequency of major discrepancies in our study. Previous studies come to different results when regarding the influence of gender and age on the rate of discrepancies [9, 14, 18, 22, 23]. When a difference in the frequency of discrepancies was detected, more discrepancies were found in older patients and in women [14, 22, 23]. In addition to gender and age, a preceding immunotherapy was analyzed for its influence on the occurrence of major discrepancies. As this kind of therapy is relatively new and not all side effects may be totally understood, it would not have been surprising if in patients who received an immunotherapy, a higher rate of discrepancies could have been noticed. This, however, was not the case. The number of patients who underwent an immunotherapy before death was relatively low (*n* = 7) though.

The selection of patients might serve as a possible explanation for the high number of major discrepancies in our study. Autopsies of oncological patients came to the focus of interest for research purpose in the 1990s. Such research autopsies aim to elucidate questions on tumor metastases and evolutionary biology of cancer, among others [24]. Regarding clinical autopsies, there are only few studies dealing with postmortem examinations in oncological cases and most of them only included cancer patients who died at an intensive care unit. Major discrepancy rates of 21–59% were reported in this collective [12, 25] which is more or less comparable to the rates of our study. At the end of a cancer patient’s life, the extent to which diagnostic procedures are used might be smaller compared to other collectives. Nevertheless, the detected high rates of major discrepancies should at least make clinicians sensitive for the risk of overlooking relevant diagnoses. On the other hand, every case has to be considered separately and in the interest of the patient, invasive procedures should sometimes be avoided.

Our data emphasize the persisting value of postmortem investigations. Despite an obviously continuously improving diagnostic machinery, important clinical aspects remain undetected in a considerable rate of cases. Moreover, our data show that a significant increase in autopsy rates is achievable by intensive and structured cooperation between clinicians and pathologists and increasing the motivation of the physicians in obtaining consent for an autopsy.

## Data Availability

Data will be made accessible upon figshare.com.
